# Application of single-cell genomics in cancer: promise and challenges

**DOI:** 10.1093/hmg/ddv235

**Published:** 2015-06-25

**Authors:** Quin F. Wills, Adam J. Mead

**Affiliations:** 1 Weatherall Institute of Molecular Medicine, University of Oxford, John Radcliffe Hospital, Oxford OX3 9DS, UK,; 2 Wellcome Trust Centre for Human Genetics, University of Oxford, Roosevelt Drive, Oxford OX3 7BN, UK and; 3 NIHR Biomedical Research Centre, Churchill Hospital, Oxford OX3 7LE, UK

## Abstract

Recent advances in single-cell genomics are opening up unprecedented opportunities to transform cancer genomics. While bulk tissue genomic analysis across large populations of tumour cells has provided key insights into cancer biology, this approach does not provide the resolution that is critical for understanding the interaction between different genetic events within the cellular hierarchy of the tumour during disease initiation, evolution, relapse and metastasis. Single-cell genomic approaches are uniquely placed to definitively unravel complex clonal structures and tissue hierarchies, account for spatiotemporal cell interactions and discover rare cells that drive metastatic disease, drug resistance and disease progression. Here we present five challenges that need to be met for single-cell genomics to fulfil its potential as a routine tool alongside bulk sequencing. These might be thought of as being challenges related to samples (processing and scale for analysis), sensitivity and specificity of mutation detection, sources of heterogeneity (biological and technical), synergies (from data integration) and systems modelling. We discuss these in the context of recent advances in technologies and data modelling, concluding with implications for moving cancer research into the clinic.

## Introduction

Massive parallel sequencing of cancer genomes has delivered major advances for our understanding of the somatic driver mutations underlying the pathogenesis of neoplastic disease (1). This knowledge has already translated through to clinical benefit in many different tumour types for diagnosis, prognostic risk stratification, targeted therapy and minimal residual disease (MRD) monitoring. It has also long been recognized that tumours evolve through serial acquisition of these somatic driver mutations through an often highly complex process of genetic diversification and clonal selection (2,3). Moreover, definitive characterization of the resulting intratumoural clonal heterogeneity is widely recognized to be a central requirement for precision medicine in haematology and oncology (2). Although cancer genome studies typically analyse genomic DNA derived from millions of cells, thereby generating data representing the average across a tumour population, computational approaches can nevertheless be used to derive clonal architecture and infer phylogenetic trees for each tumour (4,5). This approach has provided fundamental insights into how tumours clonally evolve during disease progression and under the selective pressure of therapy (4,6).

While bulk analysis is undoubtedly informative for the understanding of clonal heterogeneity of tumours, such studies are also associated with important limitations that are difficult to overcome through refined technical or computational approaches. In essence, these limitations are founded in the failure of cell population-based analysis to fully reconstruct all aspects of clonally complex tumour specimens containing highly heterogeneous populations of cells. This becomes particularly important when considering low-level subclones that might propagate subsequent disease relapse/progression. As an example, ∼1000X sequencing data are required to detect 99% of mutations carried by a 1% tumour-mass subclone analysed at the bulk level (5). Although such depth of sequencing is certainly possible, it is way beyond the depth obtained in most studies, and alternative approaches are also required. Recent advances in single-cell genomics are opening up unprecedented opportunities to definitively unravel such cellular heterogeneity in clonally complex tumours. Specific methods for single-cell genomic analysis have been recently reviewed in detail elsewhere (7), some of which are summarized in Table 1. In this review, we outline how these technical advances might be applied to address fundamental questions in cancer biology, and the key challenges that must be overcome for this pioneering technology to reach its full potential in the cancer field.

**Table 1.** Current single-cell genomics techniques

	Spatial resolution	Temporal (of the same cell)	Number of molecular features measured	Scale (number of cells)	Sensitivity for mutation detection	False positives	References
DNA							
MDA	No	No	++++	++	++	++	(8,9)
MALBAC	No	No	++++	++	+++	+++	(10)
DNA-FISH	Yes	No	+	++	++++	+/−	(11)
MIDAS	No	No	++++	++	+++	++	(12)
RNA							
Plate-based RNA-seq	No	No	+++	++	? (if whole transcript)	?	(13–18)
Microfluidics RNA-seq	No	No	+++	+++	? (if whole transcript)	?	(19)
Droplet-based RNA-seq	No	No	+++	++++	?	?	(20–22)
*In-situ* sequencing	Yes	No	++	+++	?	?	(23–25)
RNA-FISH	Yes	No	+	+++	++	+/−	(26)
Epigenetic							
Methylation	No	No	+++	++	N/A	N/A	(27,28)
ATAC-seq	No	No	++	+++	N/A	N/A	(29)
Hi-C	No	No	++	++	N/A	N/A	(30)
Mass cytometry	Yes	No	+	+++	N/A	N/A	(31,32)
Live cell imaging	Yes	Yes	+	+	N/A	N/A	(33)

## The Promise of Single-Cell Genomics in Cancer

**Figure 1.**
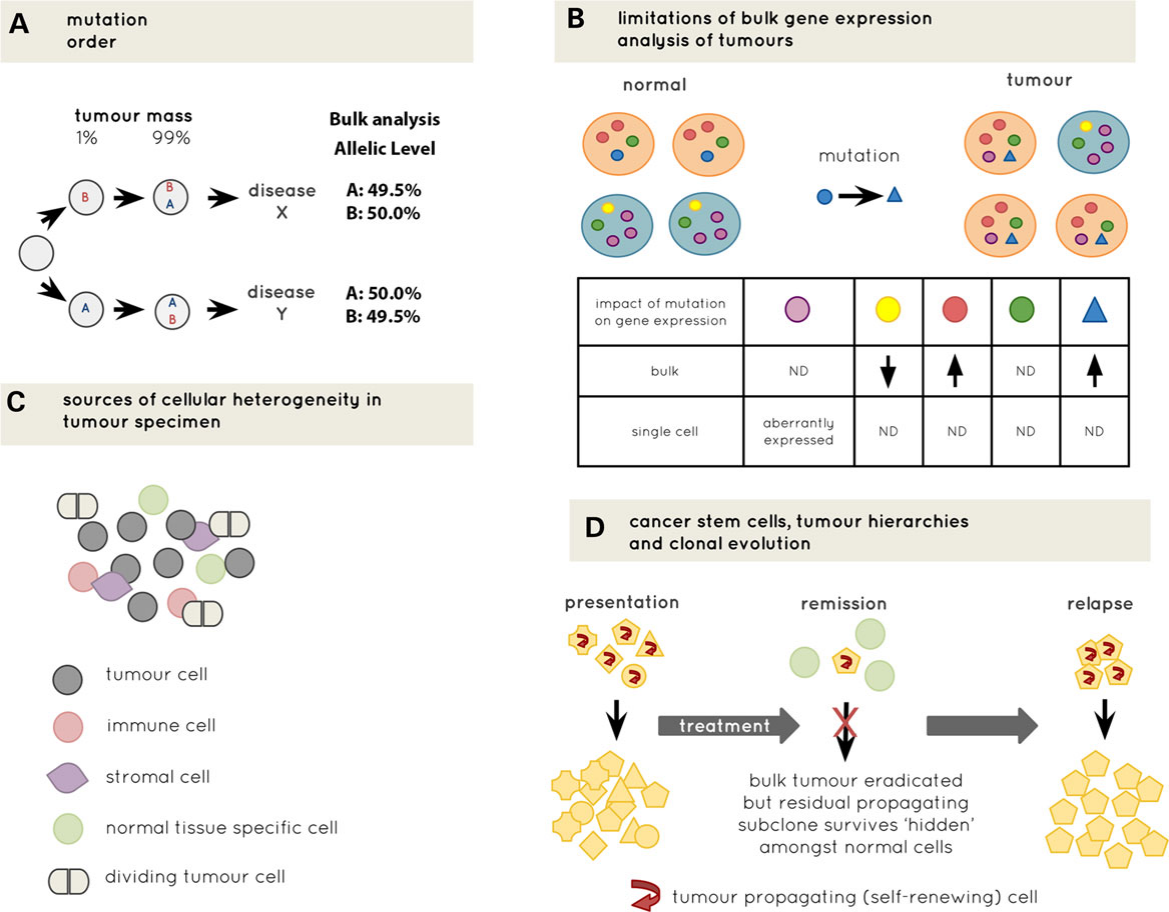
Advantages of single-cell analysis. (**A**) Diagrammatic illustration of different consequences of mutation order on disease phenotype. Cells informative for mutation order may be very rare within tumours (1% in this example) and bulk sequencing is unlikely to have sufficient resolution to determine mutation order as reads for each mutation (A and B) will be almost identical for mutations with a very similar allelic level. (**B**) Comparison of single cell versus bulk gene expression analysis. Different coloured cells (orange and blue) represent different cell types. Different coloured spots within cells represent expression level of different genes. Acquisition of a somatic mutation in the blue gene causes an expansion of orange cells. The table shows the distinct gene-expression differences detected by single cell and bulk analysis. (**C**) Heterogeneity of cellular composition of tumours that would be lost through bulk analysis. (**D**) Diagrammatic illustration of hierarchical organization within tumours throughout the disease course. Yellow indicates tumour cells, green non-tumour cells and different shapes represent different subclones of cells. This hierarchical and clonal complexity would be lost through bulk analysis. ND indicates no difference.

The most obvious application of single-cell genomics in cancer research is to define clonal architecture of tumours. For example, single-cell analysis can theoretically facilitate the detection of very low-level tumour clones with only ∼200 cells required to reliably detect 1% tumour-mass clones (34). However, the potential advantage of single-cell analysis goes far beyond this improved resolution for the detection of low-level subclones. For example, the independent acquisition of the same combination of mutation(s) in separate subclones during disease pathogenesis can occur, resulting in ‘convergent’ pathways of evolution within a tumour (11,35). The order of acquisition of mutations can also be contingent on the presence of other mutations through epistatic interactions (2). Moreover, the order of acquisition of the same combination of collaborating mutations can also influence the resulting disease phenotype (36). At the bulk population level, it might not be possible to reconstruct the tumour phylogenetic tree with this degree of resolution as cells that are informative for ancestral clones might be extremely rare within the bulk tumour (Fig. 1A). Such definitive reconstruction of phylogenetic trees is becoming increasingly important, particularly in the light of the failure of many targeted therapies to offer anything other than a minor overall survival benefit (37), which might relate to the requirement for targeting of driver mutations that are present in all the malignant cells in order to maximize efficacy (2).

Despite the promise of single-cell genomic DNA analysis in cancer, proof-of-principle studies are only just beginning to emerge in small patient numbers, and the technology has yet to enter routine translational cancer research or clinical practice. Using a variety of whole genome amplification approaches combined with sequencing or array-based copy number analysis (Table 1) (7), a number of studies have recently illustrated how single-cell genomic analysis can be applied to provide novel insights into clonal architecture, phylogenetic trees and the dynamics of mutation acquisition in breast cancer (38–41), myeloproliferative neoplasms (42), renal cell carcinoma (43), bladder cancer (44), colon cancer (45,46) and acute myeloid leukaemia (47). For the most part, these studies have illustrated how single-cell genotyping validates the major mutation clusters identified through population-based analysis while also providing additional insights into ancestral lineage and clonal complexity of tumours. Single-cell genomic analysis of circulating tumour cells (CTCs) have also documented shared mutational profiles between CTCs, primary and metastatic disease, though with insufficient power to rule out pervasive false positives for CTC-specific mutations (46,48–50). Together, these studies nicely illustrate the promise of single-cell mutation analysis in cancer to provide information about clonal heterogeneity in tumours above and beyond that provided by bulk analysis, an important step towards precision medicine.

The advantages of single-cell analysis become particularly important when considering functional genomic studies beyond mutation detection alone, e.g. gene expression or epigenetic analysis (7,51–54). The vast majority of gene-expression studies in cancer have analysed tumour cells at the population level. A major confounding factor for such analyses is that changes in the composition of heterogeneous cell populations might be falsely interpreted as a direct impact of a mutation on gene expression, a problem that can be overcome using single-cell approaches (Fig. 1B). Importantly, single-cell analysis also provides information about aberrant co-expression of genes that is lost at the bulk level (Fig. 1B). Such an approach can also, in parallel, provide information about other non-malignant stromal, immune and tissue-specific cells (43) contained within the tumour specimen (Fig. 1C). Interactions between malignant cells and multiple other components of a tumour are widely recognized to be important for cancer biology (55). Thus, a single-cell approach can help to definitively reconstruct the cellular composition of tumours as has been effectively carried out for multiple normal tissues using single-cell transcriptomics techniques (20,56–61). However, to date, only a few studies have applied whole transcriptome methodologies in the cancer field. These have primarily been proof of principle studies to assess intra-patient transcriptional diversity in primary tumours and CTCs (13,62–64), and the emergence of drug-resistance phenotypes (65). Single-cell epigenetic analysis is also emerging as an exciting new technology (27–30,66), but as yet these approaches have not been widely used in cancer.

With recent advances in therapeutic approaches for many cancers, including the advent of targeted therapy, the challenge for many tumours is often not to achieve a remission in the patient but rather to understand which cells are selectively resistant to the treatment and remain after the treatment is completed, as these are the cells that ultimately propagate disease relapse. In relation to this, it has long been recognized that some tumours are organized hierarchically and only certain populations of cells are capable of propagating a tumour, so called ‘cancer stem cells’ (Fig. 1D) (67). However, gene-expression changes in tumour initiating/propagating cancer stem cells, which can be rare in the tumour hierarchy, will be lost when a tumour is analysed at the bulk level. There is now definitive evidence supporting the existence of rare and distinct cancer stem cells in certain malignancies (67,68), and that these cells can be both rare and selectively resistant to treatment (67,69). Single-cell analysis has the unique potential to selectively analyse these rare cancer stem cells both at diagnosis and, importantly, to dissect residual cancer stem cells from normal tissue counterparts when the patient is in remission (Fig. 1D). Single-cell approaches can also be used to detect ancestral ‘pre-malignant’ stem cells, as has recently been demonstrated in AML (70). It is also apparent that cancer-associated mutations are gradually accumulated with age, as described primarily in pioneering studies in haematopoiesis (71–73). Once again, single-cell-based detection of these pre-malignant clones might be important for predicting cancer risk before the development of overt disease.

While the above studies provide proof of principle for the application of single-cell genomics approaches in the cancer field, the broader application of this pioneering technology requires a number of key challenges to be overcome. These might be summarized as those related to samples, sensitivity and specificity of mutation detection, sources of heterogeneity, synergies (from data integration) and systems modelling. Careful attention to all these challenges is required in order for single genomics to become a driving technology in cancer systems biology.

## Samples

The first step in any single-cell analysis is to develop a robust and unbiased method for the isolation of single tumour cells while minimizing loss/degradation of their genomic content. Leukaemias and other liquid tumours have obvious advantages in this regard as single cells can be isolated into individual reaction chambers using well-validated fluorescence activated cell-sorting (FACS)-based purification. However, even when using FACS-based approaches, the potential impact of sample handling on the tumour cells should not be underestimated. In solid tumours, the tissue processing required for isolation of single cells is more challenging. Some of the recent single-cell genomics studies in cancer have analysed nuclei that were sorted by flow cytometry (38,74). This process involves macrodissection of tumour from distinct anatomical locations followed by fine mincing of each tumour section in a lysis buffer (38), with FACS-based purification of single nuclei. This approach has the limitation that micronuclei (75) and cytoplasmic mRNA are lost, thereby significantly limiting the broader applicability of this technique. This can be overcome by generating suspensions of enzymatically dispersed whole single cells which can then be isolated manually (10), or by FACS or microfluidic approaches (43,63,76–78). All these methods introduce assumptions and biases that might result in selective loss of certain populations of cells based on cell size, surface antigen expression or biophysical properties. This is particularly important as cells contained within a tumour are highly heterogeneous for size, shape and phenotype (Fig. 1C) and exclusion of any cells based on any of these parameters might result in loss of cells of interest, a consideration that becomes most prominent when dealing with extremely rare cells within a tissue such as CTCs within the blood. Furthermore, the most fundamental requirement for single-cell analysis is to be able to reliably isolate a contamination-free ‘single’ cell for downstream analysis. Doublets can frequently occur with FACS and microfluidic-based single-cell isolation, as suggested through species mixing experiments (20), highlighting the need to carefully validate true single-cell capture before subsequent analysis. To avoid contamination, which can easily be introduced with the high-level amplification required for most protocols, special care is required with restricted clean rooms for single-cell analysis with regular decontamination (10).

Tumour clones evolve dynamically in both space and time; however, the above approaches are all limited by loss of this key information (Table 1). For example, a single sample from an individual tumour might reveal mutations which appear to be clonally dominant, but are then shown to be absent from other regions of the tumour (79–81). While multiple anatomically distinct biopsies of the same tumour can help with spatial information, this remains a major limitation where most single-cell approaches offer little benefit above cell population-based genomic studies. Furthermore, analysis of single cells in suspension results in loss of information with regards to direct cell-to-cell contact made by tumours, which is likely to be critical for the understanding of niche-related tumour cell interactions (82). Laser-capture microdissection can partially overcome this, but this approach is low throughput and it is also difficult to capture all of the cytoplasm or nucleus of a cell using this technique for transcriptome or DNA analysis, respectively (83). Advances in *in situ* sequencing and imaging techniques perhaps offer the best opportunity to conduct genomic analysis of tumours with definitive spatial resolution (23–25). Using a highly innovative approach, Lee *et al.* (23) were able to sequence RNA directly *in situ* in several fixed tissues. More recently, Achim *et al.* (24) and Satija *et al.* (25) presented approaches to map single-cell RNA-seq data to binarized RNA *in situ* hybridization images of marker genes. While these approaches offer the potential for spatial resolution of single-cell genomic analysis of tumours, they are all currently too early in development for broad application. Serial sampling of the same patient can provide information about evolution of tumour in time. While this is possible for liquid tumours (84,85), it is more problematic for solid tumours where sequential analysis is likely to be at the time of repeat biopsy following disease relapse/progression. Furthermore, to track ‘the same cell’ in time requires live cell-imaging approaches (33) which have not, yet as, been widely applied in the cancer field.

A further challenge with sample preparation relates to scale of analysis. Cancer stem cells and subclones might be relatively rare within the total tumour population and, unless the cells analysed are enriched on the basis of assumptions about their phenotype, large numbers of cells might be required in order to reliably detect these cells. This is particularly an issue when considering MRD detection, or isolation of CTCs (86). Many current single-cell genomics approaches are relatively low throughput, but innovative new approaches using bead-based barcoding combined with cell isolation in microfluidic droplets looks set to transform the scale for genomic analysis of single cells in cancer (Table 1) (20–22).

## Sensitivity and Specificity for Mutation Detection

While the scale of analysis of tumour cells is certainly important, it is also critical to maximize the information that is retrieved from each cell. In relation to genome-wide DNA analysis for mutation and copy number profiling, two whole-genome amplification (WGA) approaches have proved popular for single-cell DNA-seq: multiple displacement amplification (MDA) (9) and the multiple-annealing, looping-based amplification-based cycle method (MALBAC) (10). Technical details of these methods have been reviewed elsewhere (7,87,88). Choice of method depends largely on the question of interest. Each method is associated with artefacts introduced due to allelic drop out (ADO), preferential amplification of certain genomic sites and false discovery of mutations due to amplification or sequencing errors (7). In general, MDA exhibits higher fidelity in comparison with MALBAC (10). Conversely, MDA false-negative rates are greater due to lower genome coverage and low uniformity of coverage. Rates of ADO vary greatly, with rates as low as 1% reported for MALBAC and as high as 65% for MDA (10), although ∼10% ADO can probably be expected on average for most samples (42,89). In view of this, MALBAC has been the method of choice for copy number aberration, but it is important to note that reproducibility of results for smaller copy number abnormalities remains low (10,74).

In addition to false negatives in single-cell analysis due to ADO, false discovery of mutations is also a major concern. Distinguishing true somatic mutations from WGA artefacts and germline variants is clearly a fundamental requirement for single-cell mutation detection in cancer. Each WGA method has imperfections in relation to this (7) and a number of questions remain. For example, the false discovery rate was 2.5 × 10^−5^ in a study using MDA (42) as opposed to 4 × 10^−5^ with MALBAC (10), although larger differences have been reported when the two methods are compared side by side (90). Whether there is any bias for false discovery in relation to genomic region or base type remains incompletely characterized (7,42). Ultimately, it is likely that integration with data derived from single-cell mutation detection with bulk analysis (42) and validation using targeted single-cell mutation detection will help to resolve some of these issues. In summary, even with low mutation rate cancers, the high error rates with current chemistries are important factors to overcome in order to maximise the power of these methods to detect rare clones and reconstruct tumour phylogenetic trees.

Microfluidics offer the potential advantage of capturing cells within nanofluidic chambers that might improve sensitivity for mutation detection by minimizing ADO (12,91–94). An alternative approach that has been used is to amplify clones of cells, derived from a single cell, to increase the sensitivity for mutation detection by increasing the amount of starting material (36,68). This method, however, introduces an important bias that only cells which are capable of generating colonies in the selected culture conditions can be analysed. Targeted single-cell mutation detection is also likely to reduce error rates, but this methodology necessitates knowledge of the specific mutations carried by a particular tumour. For example, fluorescence *in situ* hybridization (FISH) techniques have a high sensitivity for the detection of copy number abnormalities and translocations allowing single-cell analysis in a relatively high-throughput manner. The false positive rates for this technique are of the order of 1–2%, or even less for fusion gene detection (11). Such FISH analysis has been used to reveal the clonal architecture and facilitate the assembly of ancestral trees in childhood acute lymphoblastic leukaemia (11). Similarly, PCR-based approaches can also be adapted for targeted single-cell mutation detection when the important driver mutations are known (95).

In summary, while impressive progress has been made with single-cell DNA sequencing, major efforts to minimize ADO (false negative results) and sequencing errors (false positive results) as well as comprehensive cross-comparisons of available platforms will be necessary (90) to achieve maximum benefit. How bulk and single-cell sequencing could be combined to best inform each other is also an important question and the need for these types of data integration is further discussed in what follows.

## Sources of Heterogeneity

**Figure 2.**
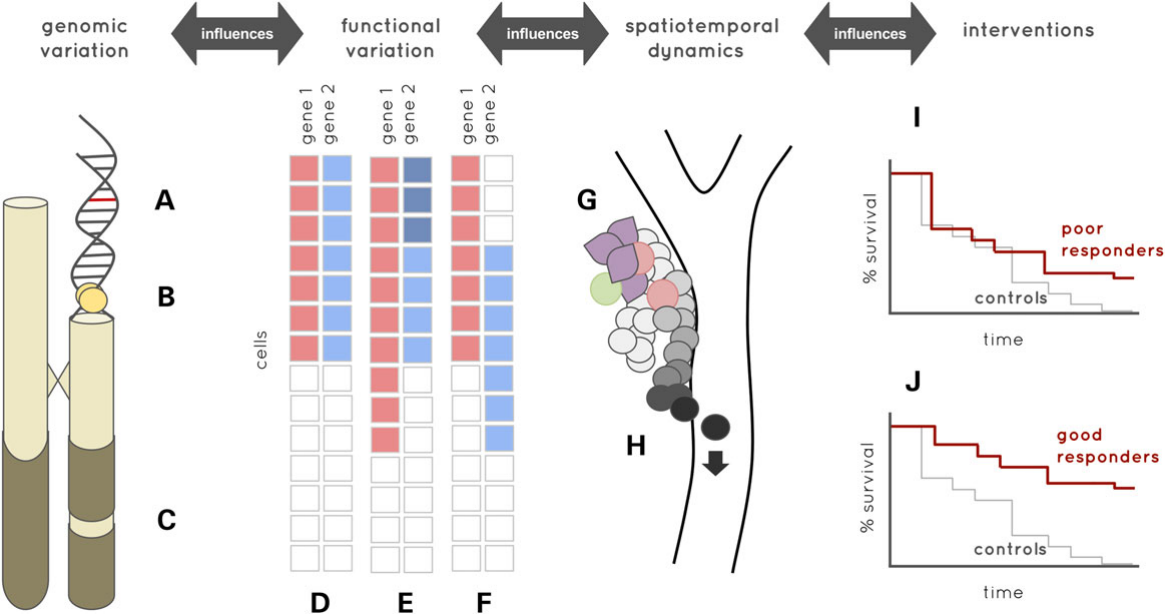
Cancer systems genomics. Modelling cancer intra- and inter-patient heterogeneity requires four levels of information, the first being high-resolution estimates of (**A**) genetic, (**B**) epigenetic and (**C**) structural variation both in germline and cancer cells. This is complemented by integration with high-resolution estimates of functional variation, such as the example gene-expression heatmaps in samples (**D–F**). Cells in sample D form two clusters, based on low-level gene expression (shown as red and blue squares) and undetectable expression (shown as white squares). The genes in sample E show different patterns of altered expression. While there is an increase in the proportion of cells expressing gene 1 at a low level, gene 2 suggests a new sub-population of cells in which it is highly expressed (shown as dark blue squares). Cells in sample F cluster into the same four groups as the cells in sample E. However, this is due to differential co-expression rather than altered expression level or expression prevalence. Bulk sequencing would not be able to differentiate sample D from sample F. Spatiotemporal information during treatment is required to understand the influence of genomic variation, intervention and cell population dynamics on emergent behaviours such as drug resistance. Cell microenvironment (such as cells in colour in **G**) is thought to play a major role in most cancers, as is the plasticity of cell phenotype over time to allow distant metastases (**H**). Translating models of intra-patient heterogeneous processes into models of heterogeneous patient response, as shown by the Kaplan–Meier curves in (**I)** versus (**J)**, is the goal of precision and stratified cancer pharmacogenomics.

One of the major challenges for the application of single-cell transcriptomics in cancer is the degree of ‘noise’ in the data that is generated. This results from multiple layers of heterogeneity which can broadly be classified as ‘real’ biological variation and technical noise generated during the sampling and analysis pipeline. As shown in Figures 1C, D and 2, biological heterogeneity can derive from multiple genetic, epigenetic, demographic, environmental and cellular factors together with stochastic gene expression at the single-cell level, which together introduce extensive heterogeneity in single-cell data sets. Technical noise is introduced at all stages in the processing pipeline from sample handling, cell-isolation, reverse transcription, cDNA amplification, sequencing and analytical stages. Therefore, it is important to minimize technical sources of variation with rigorous attention to detail in the standardization of processing pipelines, including automation and consideration of the use of microfluidics approaches which have the additional advantage of reduced reaction volumes (19). It is also important to appreciate that the degree of technical noise is not independent of biological variation in the cells analysed. On the contrary, the two are closely related, e.g. in relation to the impact of cell-cycle status, cell size and RNA content of the cell. Quality control pipelines are also of considerable importance, and there is an urgent need to define appropriate quality control steps for single-cell analysis to ensure the integrity of data sets. Bulk controls are important to show that ensembles of single cells correlate with cell analysis at the population level (19), while ‘no cell’ negative controls are essential to identify background contributions to amplified product.

Analytical techniques can also be used to help distinguish biologically meaningful heterogeneous gene-expression differences from those arising from technical noise. The use of unique molecular identifiers to barcode individual transcripts (14,15) together with inclusion of RNA-standards (96) are important considerations. A major source of functional heterogeneity is cell-cycle status, which can be accounted for using computational approaches. However, in our and others’ experience, relying only on transcriptomic markers with rapidly cycling cells can prove challenging (97). This again stresses the need for more integrated data modelling strategies for the reliable identification of challenging cell populations such as stem cells, which are often characterized by quiescence (98).

## Synergies: the Integration of Data

While cancer single-cell sequencing promises greater resolution, this does not guarantee improved mechanistic understanding or prediction of therapeutic response. With the diverse array of different single-cell approaches available, efforts are now underway to integrate methods, i.e. to allow combined modality analysis, ideally from the same single cell. The particular technical challenge when extracting multiple ‘omic’ data sets from individual cells will be ensuring that the benefits from integrating diverse modalities not only outweigh the individual methods, but also the potential data quality compromises faced when harmonizing protocols. It is these necessary compromises, scalability and cost effectiveness that will drive the interplay between different techniques. Interesting questions in experimental design may, e.g. be how best to screen with bulk sequencing to inform more focused single-cell sequencing strategies, or how to use ‘non-omic’ approaches such as high-content imaging and spectroscopy to link modalities that are mutually exclusive in the same sample (99).

A natural early step has been the integration of DNA and RNA sequencing. RNA-sequencing has the key limitation that mutation detection requires relatively high-level expression of the particular mutation in all of the cells that are analysed. Current RNA-sequencing approaches require at least 10–20 copies of a transcript for reliable detection (7). Furthermore, 3′ and 5′ biases can also limit mutation detection using RNA sequencing. Thus, mutation detection exploiting DNA and RNA-sequencing from the same cell could greatly enhance transcriptome analysis in cancer. In an early study, Klein *et al.* (100) used a combination of comparative genomic hybridization and PCR-based transcriptome analysis to analyse DNA and RNA from the same tumour cell. Using embryonic stem cell and breast cancer cell lines, Dey *et al.* (101) demonstrated comparable performance of their combined genomic DNA and reverse transcribed mRNA quasilinear amplification and sequencing (‘DR-seq’) with MALBAC and CEL-seq. Their work was one of the earliest to suggest that copy-number variation affects gene-expression variability between cells. Choosing rather to separate mRNA from genomic DNA with biotinylated oligo-dT nucleic acids and streptavidin beads, Li *et al.* (102) showed increased allelic exclusion when exposing mouse embryonic fibroblasts to a chemical mutagen. In a similar strategy, Macaulay *et al.* (103) employed biotinylated SMARTer primers in lymphoblastoid and breast cancer cell lines, demonstrating correlation between aneuploidy and gene expression. Technical studies directly comparing the respective strengths of published approaches are still lacking, but this combined approach looks set to lead to important advances in the application of transcriptome analysis in cancer.

Various other single-cell functional genomic modalities have also been reported, primarily as proof-of-principle studies in cell lines. By successfully scaling bisulphite chemistry to individual cells, Smallwood *et al.* (27) and Farlik *et al.* (28) have reported pre-amplification and amplification-free bisulphite sequencing strategies that potentially allow for improved deep-sequencing coverage and less-biased low-depth coverage, respectively. Using single-cell ATAC-seq (assay for transposase-accessible chromatin with sequencing) to identify open chromatin, Cusanovich *et al.* (29) were able to cluster cell lines with a remarkably low median of 1685 reads per cell. Single-cell Hi-C (chromosome conformation capture with sequencing) has also been demonstrated in recent work by Nagano *et al.* (30), where active chromatin domains in mouse splenic cell nuclei localized to the surface of their spatial chromosome territories. ChiP (chromatin immunoprecipitation) and histone modification assays have proved more problematic (66). Integrating these functional genomic approaches with mutation and transcriptome analysis is now the challenge.

Validation of single-cell gene-expression data requires integration with different single-cell genomic approaches and also the use of single-cell protein expression analysis and functional assays. A commonly used approach is to validate RNA-sequencing data using targeted gene-expression analysis (56). Validation of gene expression at the protein level is also possible using ‘index-sorting’ of cell-surface markers and correlating this with gene expression in the same single cell (104). Other single-cell protein-analysis techniques such as mass-cytometry (31,32) do not currently allow direct integration with genomic analysis, but provide an important platform for the validation for single-cell genomic analysis including the possibility to retain spatial information (32). Finally, validation of single-cell genomic analysis in functional cellular assays is also important, e.g. *in vitro* or *in vivo* stem-cell assays, as recently employed for the normal haematopoietic system (104). As most of the techniques for genomic analysis require destruction of the cell, it is difficult to combine this approach with functional cellular assays of the same cell unless using paired daughter cell techniques where the immediate progeny of a single cell are separated for differential analysis, with the obvious caveat that the daughter cells may differ significantly between each other and the mother cell (105).

## Systems Modelling

As with other systems biologies, cancer systems biology remains largely divided between data-driven and model-driven strategies. High-throughput data approaches have predominantly worked towards associating variation in gene sequence and expression with pathology and treatment response. These ‘big data’ strategies traditionally limit themselves to integrating the types of data already discussed, with the aim of developing bottom-up multi-scale descriptions of cancer molecular biology. Conversely, mathematical modelling has more typically focused on higher-level tumour cell dynamics such as invasion, angiogenesis and metastasis. Single-cell genomics arguably provides the first scalable opportunity to begin unifying these strategies under the common goal of modelling complex system behaviours. As depicted in Figure 2, for cancer, this means models of the spatiotemporal changes in cell populations while predicting the influence of genetic drivers and therapies on these model parameters.

Such cancer ‘systems genomics’ modelling has yet to be fully realized. Single-cell studies have predominantly focused on gene- and protein-expression methods in attempts to robustly and reproducibly describe cell sub-populations, while also accounting for technical contributions to population structure. These approaches can be broadly catagorized as algorithms that project linear and nonlinear covariance structures (97,106), hierarchical and partitioning clustering algorithms (107,108) and network trajectory algorithms (109). Two important contributions to cancer cell state that have been considered in early studies are cell cycle and microenvironment. The use of transcriptional markers alone to determine individual cell-cycle states has proved challenging in rapid cycling cells, with Patel *et al.* (64) resorting to a cell-cycle signature score in glioblastoma cells to study gene coexpression and Buettner *et al.* (97) suggesting the removal of cell cycle as a latent variable based on known periodic genes.

The modelling of regulatory networks, coexpression modules and gene ‘noise’ remains currently under-explored in single-cell genomics. It is thought that—for reasons such as transcriptional bursting—most genes will demonstrate highly variable expression even in similar cells, and that this expression variability may enable the study of regulation dysfunction not possible with bulk approaches. Understanding this type of functional stochasticity may also prove as important to modelling the drivers of cancer behaviour and drug response as has been the traditional focus on DNA stability/‘noise’ (110,111). Based on our experience associating genetic variability with gene-expression phenotypes (112), current single-cell costs and scalability likely limit the power to broadly study gene-network parameters such as robustness, redundancy and degeneracy in most cancers. However, at the very least, it now seems possible to begin testing of hypotheses such as the ‘mutator phenotype’ and its relationship to functional variability, cell phenotypes and drug response (10).

## Conclusions

The field of single-cell genomics is advancing at a truly remarkable speed, and looks set to transform cancer-biology research over the coming years. The deep characterization of the clonal and functional architecture of tumours that might be delivered through single-cell analysis is of obvious relevance for the management of cancer patients through refined risk-stratification, targeted therapy selection and MRD detection. The scale of single-cell analysis is likely to increase dramatically both in terms of the numbers of cells and patients that can be handled. However, for these new technologies to fulfil their potential for precision medicine, a number of not inconsiderable hurdles remain. The next steps in the field are to address the key challenges outlined in this review and to comprehensively compare and standardize the resulting methodologies. With the speed of technical advance in this field over the last few years, we anticipate that these challenges will be overcome and we will soon enter an era where single-cell cancer genomics becomes routine.

*Conflict of Interest statement.* None declared.

## Funding

This work is supported by a Medical Research Council Senior Clinical Fellowship (A.J.M.) and the National Institute for Health Research (NIHR) Oxford Biomedical Research Centre Programme (A.J.M.). The views expressed are those of the authors and not necessarily those of the NHS, the NIHR or the Department of Health. Funding to pay the Open Access publication charges for this article was provided by a UK Research Councils Open Access Block Grant.
